# Acupuncture alleviates CSDS-induced depressive-like behaviors by modulating synaptic plasticity in vCA1

**DOI:** 10.7150/thno.106751

**Published:** 2025-03-31

**Authors:** Lin Cong, Shengkai Ding, Yu Guo, Jiamin Tian, Lu Liu, Mengqi Su, Ruilin Xie, Yinuo Wang, Tongrui Wu, Lianbin Zhao, Xiaogang Pang, Yuhong Jing, Hao Wu, Hui Shen, Yuanyuan Li

**Affiliations:** 1 Laboratory of Chinese Medicine Brain Science, Innovative Institute of Chinese Medicine and Pharmacy, Shandong University of Traditional Chinese Medicine, Jinan 250355, China.; 2 Experimental Center, Shandong University of Traditional Chinese Medicine, Jinan 250355, China.; 3 Department of Cellular Biology, School of Basic Medicine, Tianjin Medical University, Tianjin 300070, China.

**Keywords:** acupuncture, depression, LR3, fiber photometry, synaptic plasticity

## Abstract

Acupuncture (Acu) has been clinically validated as an effective treatment for depression. However, the underlying mechanism of Acu treatment's antidepressant effect remains unclear.

**Methods:** We investigate the antidepressant effects of Acu treatment at the LR3 point in mice subjected to chronic social defeat stress (CSDS). GCaMP6m-based fiber-optic photometry was employed in the ventral CA1 (vCA1) regions for the first time to monitor Ca^2+^ transients *in vivo* during behavioral testing. Electrophysiological recordings were used to detect the activity and synaptic function of pyramidal neurons. Golgi staining was performed to measure the density of dendritic spines in the vCA1. Western blot analysis was conducted to quantify the expression levels of phosphorylated CaMKIIα, AMPA receptor protein (GluA1, GluA2), and brain-derived neurotrophic factor (BDNF) in the hippocampus.

**Results:** Our findings indicated that Acu treatment significantly alleviated emotional deficits and restored the activity of pyramidal neurons, which were suppressed by CSDS. Acu treatment also reversed the decrease in spontaneous excitatory postsynaptic currents (sEPSCs), thereby enhancing glutamatergic transmission. Moreover, Acu treatment improved synaptic plasticity, as evidenced by increased dendritic spine density and restored expression levels of phosphorylated CaMKIIα, GluA1, GluA2 and BDNF.

**Conclusion:** Collectively, these findings suggest that Acu treatment alleviates depressive-like behaviors induced by CSDS and enhances synaptic function in the vCA1 region, potentially through mechanisms involving increased AMPAR trafficking and BDNF expression.

## Introduction

Depression is recognized by the World Health Organization as a significant contributor to the global disease burden 1. In contemporary society, escalating stress levels have led to an increased incidence of depression, particularly due to chronic social defeat stress (CSDS), which manifests as reduced stress resilience, anxiety, depressive symptoms, and social withdrawal behaviors 2. Current pharmaceutical treatments often come with undesirable side effects and may result in drug resistance for some patients 3. Therefore, it is essential to explore the pathogenetic mechanisms underlying CSDS-induced depression and to identify novel therapeutic strategies.

Acupuncture has demonstrated efficacy in alleviating depressive symptoms with minimal side effects 4, while antidepressant medications remain the primary treatment option 5. The Taichong point (LR3), located on the foot syncopal liver meridian, is known for its efficacy of dredging the liver, regulating qi, harmonizing emotions, and is regarded as an effective point for treating depression. Researchers have employed resting-state functional magnetic resonance imaging (fMRI) to explore the mechanisms of acupuncture at LR3, demonstrating its activation of brain networks involved in associative functions and emotional cognition 6. Notably, electroacupuncture at LR3 over a seven-day period has significantly ameliorates depression-like behaviors in a chronic restraint stress model in mice 7. However, the mechanisms of by which a single stimulation of LR3 exerts its antidepression effects remain largely unclear.

The hippocampus plays a crucial role in mood regulation and cognitive function, with its dysfunction being implicated in the pathophysiology of depression. In various chronic stress models, hippocampal pyramidal neurons exhibit apical dendritic atrophy, reduced density of postsynaptic spines and AMPA receptors (AMPARs), along with deficits in excitatory neurotransmission and long-term potentiation (LTP) 8-12. AMPARs are critical for synaptic plasticity, and abnormalities in their function or the proteins regulating their transport are associated with numerous neurological and psychiatric disorders 13. Stress reduces the expression of the GluA1 subunit of AMPARs in the ventral CA1 (vCA1) region, impairing AMPAR-mediated synaptic excitation 14. Additionally, prolonged exposure to socially frustrating stress leads to a reduction in excitatory postsynaptic currents in the prefrontal cortex and hippocampus, indicating a decline in functional plasticity at glutamatergic synapses 15-17. At the molecular level, brain-derived neurotrophic factor (BDNF) plays a crucial role in mediating synaptic plasticity, influencing neuronal morphology and physiology. It promotes neuronal growth, facilitates the formation and stabilization of synapses and enhances LTP 18. Chronic stress or early life stress significantly reduces BDNF levels in the hippocampus, leading to impaired synaptic plasticity and depression-like behavior 19,20.

In this study, we employed the CSDS model to analyze Ca^2+^ signals simultaneously recorded in the vCA1 using fiber photometry and to examine their relationship with depression-like and social behaviors. We assessed changes in synaptic transmission using electrophysiology and measured the expression levels of GluA1, GluA2, CaMKIIα and BDNF levels in the vCA1. Our results suggest that acupuncture at LR3 elicits an antidepressant response by altering pyramidal neuronal activity, increasing AMPAR trafficking to the cell membrane, and enhancing BDNF expression.

## Materials and methods

### Animals

Male C57BL/6J mice (8-10 weeks) and CD-1 retired breeder mice (28-32 weeks) (Beijing Vital River Laboratory Animal Corp. Ltd. China) were housed in SPF animal laboratory of Shandong University of Traditional Chinese Medicine. All mice were housed in a controlled environment with a 12 h light/12 h dark cycle, 50-60% humidity, and a temperature of 23 ± 2 ℃, ensuring a quiet and odor-free environment. The experiment was carried out after one week of adaptive feeding. All experiments were approved by the Ethics Committee of Shandong University of Traditional Chinese Medicine.

### Social defeat stress paradigm (CSDS)

The implementation of CSDS was performed as previously reported 21. Before modeling, male CD-1 retired breeding mice were reared in a single cage for 7 days to establish territorial awareness. At the same time, two screenings were performed with the criteria of no less than five consecutive attacks during a 3 min period and an attack latency of the initial attack must be less than 60 s. The C57BL/6J mice in the model group were placed in the cage of unfamiliar CD-1 mice and attacked by CD-1 mice for 5~10 min. Subsequently, a transparent partition with holes was used to separate for 24 h to avoid direct contact between the two, but they could still see and smell each other. This process was repeated for about 7-10 days, and C57BL/6J mice were exposed to different CD-1 mice every day, and finally showed depressive-like behavior.

### Behavior tests

**Sucrose Preference Test (SPT):** All mice were raised in single cages to adapt to sucrose water: a bottle of 1% sucrose water was placed on each side of the cage for two days. On the third day, the bottles of sucrose water were replaced with water. The mice were fasted from water and food for 24 h prior to testing. On the following day, each mouse was individually placed in a cage with two leakproof water bottles, with 1% sucrose solution on the left and water on the right. Each mouse could freely contact two water bottles. The percentage of sucrose preference was calculated using the following formula: Sucrose preference (%) = [sucrose intake (g) / (sucrose intake (g) + water intake (g))] × 100%.

**Tail Suspension Test (TST):** The tails of the mice were wrapped around the scaffold about 1cm from tail tip. The camera was adjusted to horizontally align with the mouse's body, with a detection time of 6 min. VisuTrack animal behavior analysis software was used to automatically count the immobility time of mice within the central 5 min period.

**Forced Swim Test (FST):** The mice were placed into a transparent cylinder (diameter 20 cm, height 50 cm) filled with water at 23 ± 1 ℃ throughout the experiment. The water depth was adjusted according to the animal weight, and the animal tail should maintain a certain distance from the bottom of the test box. After a 1 min acclimation period, the duration of time that the mouse remains in a stationary state within the subsequent 5 min is recorded.

**Open Field Test (OFT):** Mice were placed within a test box (40 cm × 40 cm × 25 cm). The VisuTrack animal behavior analysis software is utilized to record and analyze the time spent by the mice in both the central and peripheral zones, as well as to measure the total distance traveled by the mice within a 5 min period. Prior to each behavioral experiment involving the mice, the open field area is meticulously cleaned with 75% alcohol to ensure a clean and sterile environment.

**Social Interaction Test (SIT):** Mice were placed in an open field (40 cm × 40 cm × 40 cm) with a metal cage on one side. The test was conducted in two phases. In the initial phase, the test mouse was allowed 2.5 min to freely explore the open area in the absence of a CD-1 mouse ("no target"). The time spent by the test mouse in the interaction zone and the corner area was recorded. In the subsequent 2.5 min session ("target"), an unfamiliar aggressive CD-1 mouse was placed inside an empty cage. The duration spent in the corner and the interaction zone was monitored and measured. The social interaction index was calculated as the ratio of time spent in the interaction zone with the target present to the time spent in the interaction zone without the target.

### Acupuncture treatment (Acu treatment)

**Figure 1:**
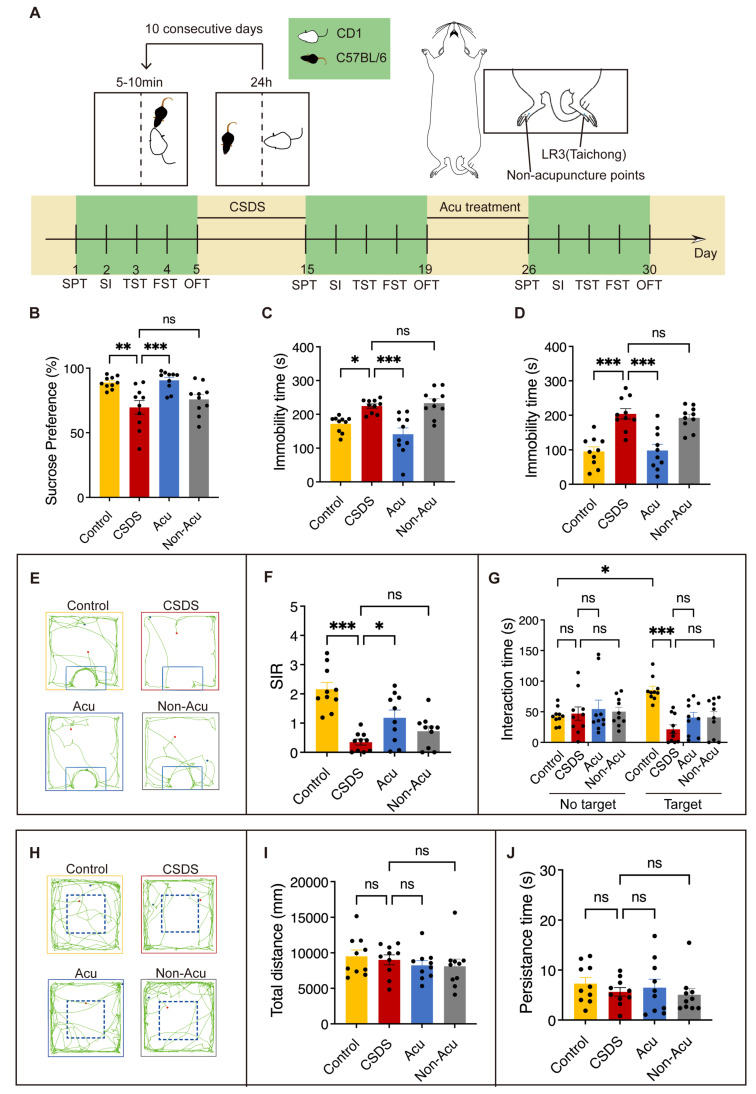
** Acu Treatment Reverses CSDS-induced behavioral disorders. (A)** A paradigm for behavioral modeling of CSDS in mice; schematic representation of therapeutic points and experimental protocol for behavioral testing. **(B)** Coefficient of sucrose preference in the SPT. **(C)** The duration of immobility in the TST for 5 min. **(D)** The duration of immobility in the FST for 5 min.** (E)** Trace diagrams for SIT. **(F)** Social Interaction Ratio for the SIT. **(G)** Time spent in social interactions “No target” and “Target” for SIT. **(H)** Representative locomotion traces in OFT.** (I)** The total distance travelled in OFT. **(J)** Persistence time in the central area of OFT. n = 10 mice/group. Panel G, two-way ANOVA with Tukey's multiple comparisons test, Remaining panels, one-way ANOVA with Tukey's multiple comparisons test. Data are represented as the mean ± SEM. *P < 0.05, **P < 0.01, ***P < 0.001. n.s., no significant difference.

Acupuncture treatments were performed once a day at the LR3 acupoints in the Acu group of mice (Figure 1). The LR3 acupoints are located in the depressions of the first and second metacarpal of the hind limbs. The control group mice received no interventions, while the Non-Acu groups underwent needle insertion at a non-acupoint location for seven consecutive days. The selected non-acupoint was located in the depression between the second and third metacarpal bones of the hind limbs. Mice were anesthetized with isoflurane (2% for induction, 1-1.5% for maintenance). Sterile, disposable acupuncture needles (Wujiang City Cloud & Dragon Medical Device Co., Ltd.; 0.16 × 7 mm) were inserted obliquely into the bilateral LR3 acupoints to a depth of 3 mm. Manual stimulation was applied every 10 min, and the needles were left in place for 30 min. Acu treatments were continued for 7 consecutive days.

### Immunofluorescence (IF)

Mice were first perfused transcardially with phosphate-buffered saline (PBS), followed by PBS containing 4% paraformaldehyde (PFA). The brains of the mice were then fixed in PFA at 4 °C for 12 h, and subsequently immersed in PBS solutions containing 20% and 30% sucrose, respectively. Coronal or sagittal slices were cut at a thickness of 40 microns using a freezing microtome (Leica, CM1950). After rinsing with PBS, the slices were incubated in blocking solution (PBS containing 5% bovine serum albumin and 0.3% TritonX-100) for 2 h. Subsequently, the slices were incubated in the blocking solution (PBS containing 3% bovine serum albumin and 0.3% TritonX-100) at 4 °C for 24 h, with the addition of the primary antibody: rabbit anti-c-Fos (1:1500, Synaptic Systems). Following this, the slices were rinsed with PBS three times for 5 min each, and then incubated with the secondary antibody (goat anti-rabbit Alexa Fluor 488, Abcam, diluted 1:1000) at room temperature for 1.5 h. Afterward, the slices were rinsed with PBS three times for 5 min each. Anti-fade mounting medium containing DAPI (Beyotime, P0131) was applied for mounting. Images were acquired using a Keyence microscope and analyzed using Fiji software.

### Electrophysiology

**Slice preparation:** Mice were rapidly decapitated, the brains was quickly dissected out after decapitation and then perfused with 20 ml ice cold solution of artificial cerebrospinal fluid (ACSF) containing (in mM): 120 NaCl, 2 CaCl_2_·2H_2_O, 2.5 KCl, 2 MgSO_4_·7H_2_O, 1.25 NaH_2_PO_4_·2H_2_O, 10 C_6_H_12_O_6_·H_2_O, and 26 NaHCO_3_, saturated with 95% O_2_, 5% CO_2_ and buffered to a pH of 7.4. Following sectioning at 300 μm thick the slices were incubated in oxygenated ACSF at 32 °C to recover and then transferred to the recording chamber.

**Recording of AMPAR sEPSCs:** Neurons in the vCA1 region of the hippocampal slice were visualized using a DIC-infrared upright microscope, and recorded using whole-cell patch clamp procedures at -70 mV. The pipette resistance was in the range of 4-6 MΩ. The intracellular solution contained (in mM): 140 K-gluconate, 2 MgCl_2_, 10 HEPES, 10 BAPTA, 2 Mg-ATP, 0.5CaCl_2_-H_2_O, 0.5 Li-GTP, pH 7.2-7.4, 280-290 mOsm. 5 mM QX-314 was added to block voltage-gated Na^+^ channels and GABA_B_ receptors. The sEPSCs were recorded in the presence of 100 μM picrotoxin (PTX).

**Recording of GABA_A_R sIPSCs:** The pipette electrodes, with a resistance of 3-5 MΩ, were filled with the internal solution containing (in mM): 140 CsCl, 10 HEPES, 2 MgCl_2_, 0.5 EGTA, 2 MgATP, 0.5 Na 3GTP, 12 phosphocreatine and 30 NMG, pH 7.2-7.4, 280-290 mOsm. 5 mM QX-314 was added to block voltage-gated Na^+^ channels and GABABRs. After the neuron was voltage-clamped at -70 mV, sIPSCs were recorded in the presence of 50 μM kynurenic acid (KYN).

**Recording of AMPA/NMDA:** As described previously 22, for whole-cell patch-clamp recording, blue light with a wavelength of 470 nm was used, with light intensity ranging from 1 to 10 mW/mm². The stimulation frequency was set at 20 Hz, and the stimulation duration was 5-20 ms. The intracellular solution contained (in mM): 135 CsMeSO₃, 8 NaCl, 10 HEPES, 0.3 Na-GTP, 4 Mg-ATP, 0.5 EGTA (pH 7.3, 290 mOsm). When recording AMPAR-EPSCs, the membrane potential was held at -70 mV, and 100 μM picrotoxin (PTX) was added to the bath solution to block GABA_A_ receptor currents. When recording NMDAR-EPSCs, the membrane potential was held at +40 mV, an amplitude of 100 ms post-stimulus was identified as the NMDA-specific response. Additionally, 5 mM QX314 was included in the intracellular solution to block intracellular Na⁺ channels.

**Recording of AP**: Recordings were obtained using borosilicate glass micropipettes (4-6 MΩ). The internal pipette solution consisted of the following (in mM): 145 K-gluconate, 10 HEPES, 1.0 EGTA, 2.0 Na_2_ATP, and 0.4 NaGTP, pH 7.2-7.4, 280-290 mOsm. Action potentials were recorded in current-clamp mode at a holding potential of -70 mV, with cells receiving a series of 15 hyperpolarizing/depolarizing current stimuli starting at -30 pA, incrementing by 5 pA, and lasting for 600 ms. The stimulus current value and amplitude of the first action potential evoked by the current stimuli were recorded.

### Virus injections

Mice were anesthetized with isoflurane (2% for induction, 1-1.5% for maintenance) and placed in a stereotaxic apparatus (RWD Life Technology, China). A volume of 200 nL rAAV-CaMKIIα-GCaMp6-WPRE-hGH polyA or rAAV-hSyn-hChR2 (H134R)-mCherry-WPRE-hGH polyA (Brain VTA Co., Ltd, Wuhan, China) were injected into the vCA1 (from bregma: anterior-posterior (AP), -3.28 mm, mediolateral (ML), 3.25 mm, and dorsal-ventral (DV) from the dura, -3.83 mm). All injections were performed at a speed of 40 nL/min. After each injection, the syringe was left for 10 min before being withdrawn to ensure the virus fully diffused. Mice recovered on a heating pad until normal behavior resumed.

### *In vivo* fiber photometry recording

Fiber photometry was used to record calcium signals from excitatory pyramidal neurons in the vCA1, using a three-color single channel optical fiber recording system (Thinker Tech, Nanjing). An optical fiber (ID:200 μm, OD:1.25 mm, FiberMD 200 μm, NA0.37, Shanghai Fiblaser Technology Co.,Ltd) was implanted into 0.2 mm above the injection site. Three weeks after virus injection and fiber implantation, neuronal activity and behavioral responses were synchronously recorded During the TST and novelty-suppressed feeding test (NSFT). Behavioral videos were recorded using Visu Track (XinRuan Co.Ltd, Shanghai). The 470 nm and 405 nm laser were used for GCaMP6m signal and autofluorescence measurement, respectively. The 405 nm channel served as a control and was subtracted from the GCaMP6m channel to eliminate autofluorescence, bleaching and motion effects. Fluorescence changes (Z-score = (F - F0) / F0) were calculated and analyzed using custom-written MATLAB codes (MATLAB R2017b, MathWorks). F0 was defined as the mean baseline signal from -5 s to 0 s or -3 s to 0 s. The area under the curve (AUC) values and peak were calculated across signal time-bins. Following recordings, mice were transcardially perfused with 4% PFA, and brain tissues were processed to confirm viral expression and fiber optic placement. Data were retained for analysis only if the viruses and fibers were localized to the correct target regions.

### Golgi-Cox staining

Golgi impregnation of whole brains was accomplished sing the FD Rapid Golgi Stain kit (FD NeuroTechnologies, PK401, USA). Coronal sections of 100 μm thickness were obtained using a vibratome (Leica VT1200S). The neurons were viewed with a 100x oil objective on an OLYMPUS IX73 upright light microscope. Tertiary or lesser order apical dendrites (≥ 10 μm) were chosen to determine spine density, the dendrites should be paralleling to imaging focal plane to the full extent for easier and better processing. Identification of spines was accomplished semi-automatically using ImageJ (https://imagej.nih.gov/ij/). For dendritic number, we counted basal branches emanating from the cell body manually. Spine density assessment were done under double-blind conditions.

### Western blotting

Proteins from hippocampus were extracted and protein concentration was determined by BCA method. 40 µg of protein was used for electrophoresis. The proteins were then separated by polyacrylamide gel electrophoresis and transferred onto a polyvinylidene fluoride (PVDF) membrane. After blocking the PVDF membrane with 5% skimmed milk powder for 2 h, p-GluA1 (Invitrogen, MA5-27975), p-GluA2 (Ser880) (absin, abs147627), p-CaMKIIa (Thr286) (Cell Signaling, 12716), BDNF (Thermo Scientific, 710306) and β-actin (Cell Signaling, 4970) were added as primary antibodies, and incubated at 4 ℃ overnight. Dilute the secondary antibody (Protein-tech, SA00001-2) and add to the incubation box. Incubate for 1.5 h at room temperature. The immunoblot was quantified using a very sensitive ECL chemiluminescent solution. Gray scale values of protein bands were evaluated using Image J software. β-actin was used as an internal standard.

### Data analysis and statistics

All experiments and data analyses were conducted blindly. The number of experimental replicates (n) was indicated in the figure legends and referred to the number of experimental subjects independently treated in each experimental condition. Heatmap was plotted by https://www.bioinformatics.com.cn, an online platform for data analysis and visualization. GraphPad Prism 10 was used to conduct statistical analyses. The one-way or two-way analysis of variance (ANOVA), Kolmogorov-Smirnov test were used appropriately (Supplementary Table 1). Specific statistical methods and post hoc tests are described in the relevant figure legends. Statistical significance was set at n.s., no significant difference, *P < 0.05, **P < 0.01, ***P < 0.001, ^##^P < 0.01, ^###^P < 0.001. Data were presented as mean ± SEM.

## Results

### Acu treatment reverses CSDS-induced behavioral disorders

Previous studies have demonstrated that CSDS induces depressive and anxiety-like behaviors, as well as social impairments in mice 23. To examine the effect of Acu treatment on CSDS-induced behavioral disorders, mice were subject to a well-established CSDS model for 10 consecutive days, followed by several behavioral tests. Subsequently, we performed acupuncture treatment for 7 consecutive days (Figure 1A). CSDS significantly reduced the sucrose preference index in the sucrose preference test (SPT) (Figure 1B) and increased immobility time in both the tail suspension test (TST) (Figure 1C) and forced swim test (FST) (Figure 1D). Mice exposed to inescapable aversive environments like the TST and FST initially display vigorous escape attempts but eventually transitioned to a passive coping (PC) state 24. Acu treatment significantly reversed these behavioral impairments (Figure 1C, D) and maintained its effects over an extended period (Figure S1A-C). These results indicated that Acu treatment induces antidepressant effects in CSDS-exposed mice.

To further investigate whether Acu treatment improved CSDS-induced social avoidance, we performed the social interaction test (SIT). CSDS significantly decreased the social interaction ratio (SIR), which was effectively restored by Acu treatment (Figure 1E, F) and sustained over a prolonged period (Figure S1D). Moreover, the time spent in the interaction zone during the SIT without the target showed no discernible differences among the four groups. However, when the target was present, Acu treatment did not fully restore socialization in CSDS mice. Although the overall social behavior was not completely normalized, there was a noticeable trend towards increased time spent in the interaction zone for the Acu-treated group compared to the untreated CSDS group (Figure 1E, G). No significant differences were observed in the total distance of the open field test (OFT) (Figure 1H, I), indicating that neither CSDS nor Acu treatment affected the motor capacity of the mice. In addition, the time spent in the central zone of the OFT also showed no significant differences across groups, suggesting the mice did not exhibit a significant anxiety-like state (Figure 1H, J). Together, these results demonstrate that Acu treatment significantly reversed the social interaction deficits induced by CSDS.

### Acu treatment enhances intrinsic excitability of vCA1 pyramidal neurons

**Figure 2:**
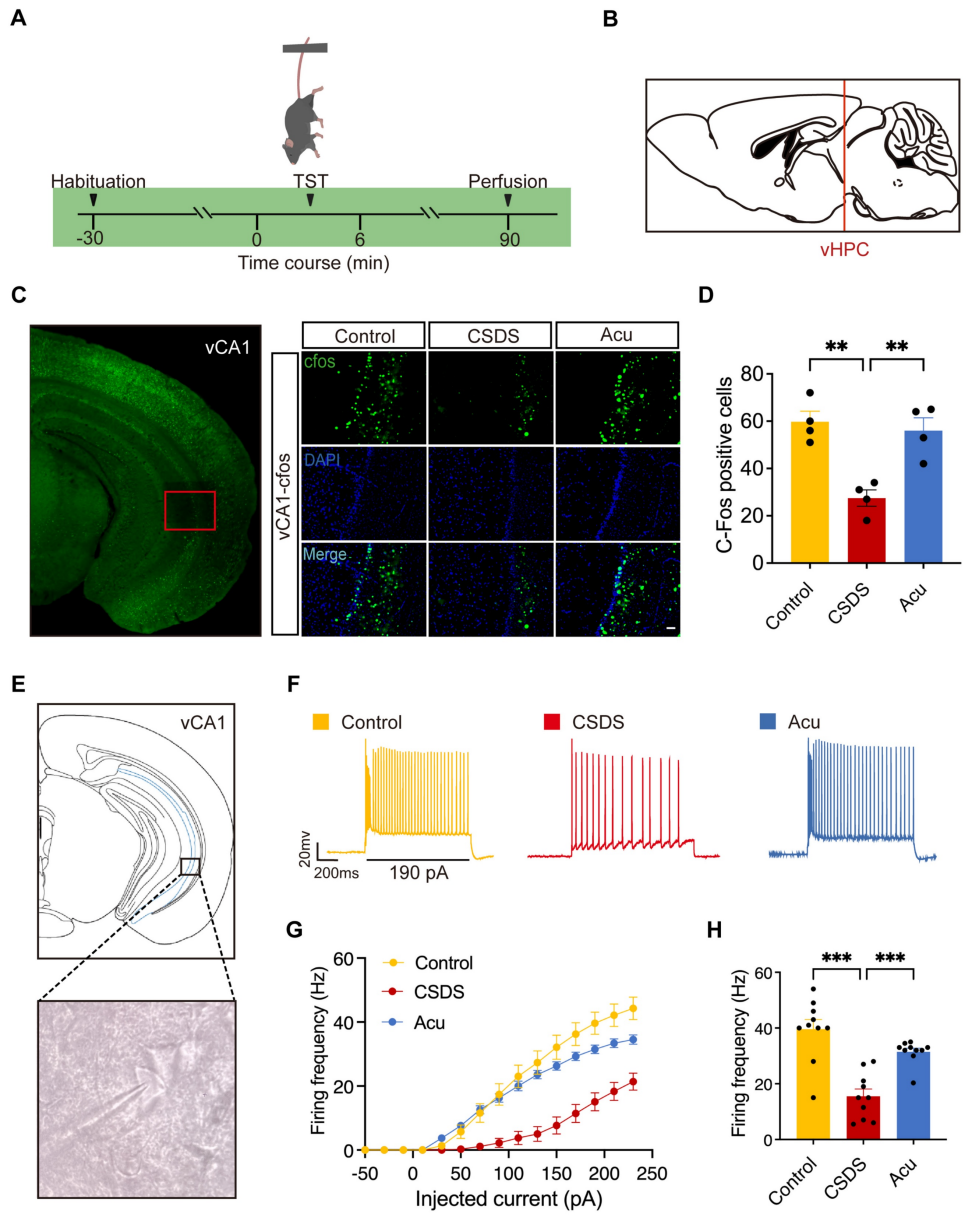
** Acu treatment enhances the pyramidal neuronal activity of vCA1. (A)** Experimental diagram of c-Fos staining. **(B)** Location of selected sagittal slices of the ventral hippocampus in the mouse brain atlas.** (C)** The c-Fos expression in vCA1 area. Scale bar, 50 μm. **(D)** Statistical results of c-Fos positive cells (one-way ANOVA with Tukey's multiple comparisons test). n = 4 mice/group. **(E)** Example of membrane-clamp recording of vCA1 pyramidal neurons in whole-cell configuration. **(F)** Example voltage traces evoked by 190 pA inward current injection in vCA1- pyramidal neurons. Scale bars, 400 ms, 20 mV. **(G)** Summarized AP data recorded from vCA1-pyramidal neurons; 0~230 pA, 20 pA steps, 1 s duration. **(H)** Spontaneous firing rates. n = 10 neurons from 3 mice, respectively. One-way ANOVA with Tukey's multiple comparisons test; Data are represented as the mean ± SEM. **P < 0.01, ***P < 0.001.

Previous studies have demonstrated that Acu treatment reduces neuronal apoptosis in hippocampal regions and improves behaviors associated with pain and depression in rats 25. However, the specific hippocampal regions involved in the neuromodulatory effects of Acu treatment in depression require further exploration. To clarify the regions through which Acu treatment mediates its antidepressant effects in CSDS mice, we fluorescently labeled brain slices with c-Fos, a marker of neuronal activation, following the TST (Figure 2A). This approach allowed us to map c-Fos expression across various hippocampal regions and assess their activation following CSDS and Acu treatment (Figure 2B). CSDS mice exhibited a dramatic decrease in c-Fos expression in the vCA1, indicating reduced neuronal activation. Acu treatment significantly increased c-Fos expression (Figure 2C, D), suggesting enhanced neuronal activity. However, there was no significant difference in c-Fos expression between the CSDS and Acu-treated groups in the dorsal CA1 (dCA1) (Figure S2) and DG (Figure S3). These findings suggest that the vCA1 region of the hippocampus is specifically involved in the neuromodulatory effects of Acu treatment, potentially contributing to its ability to regulate depressive-like behaviors in CSDS mice.

To further investigate the effects of Acu treatment, we performed whole-cell patch-clamp recordings in pyramidal neurons from the vCA1 region of the hippocampus (Figure 2E) to assess action potential (AP) firing (Figure 2F). CSDS significantly reduced the firing frequency of these neurons, indicating impaired intrinsic excitability. Following Acu treatment, the firing rate was restored to levels comparable to those in control mice (Figure 2F- H). This restoration of neuronal excitability was not associated with changes in resting membrane potential (RMP) (Figure S4A), membrane resistance (Rm, Figure S4B) or membrane capacitance (Cm, Figure S4C). Although the CSDS model did not change the threshold (Figure S4D), it increased the rheobase of pyramidal neurons. Acu treatment restored the rheobase to the level of control mice, increasing the excitability of pyramidal neurons (Figure S4E). These findings suggest that Acu treatment reverses the CSDS-induced decrease in the intrinsic excitability of vCA1 pyramidal neurons, which may contribute to its antidepressant effects.

### Acu treatment enhances vCA1 excitatory neurons activity during TST and NSFT

**Figure 3:**
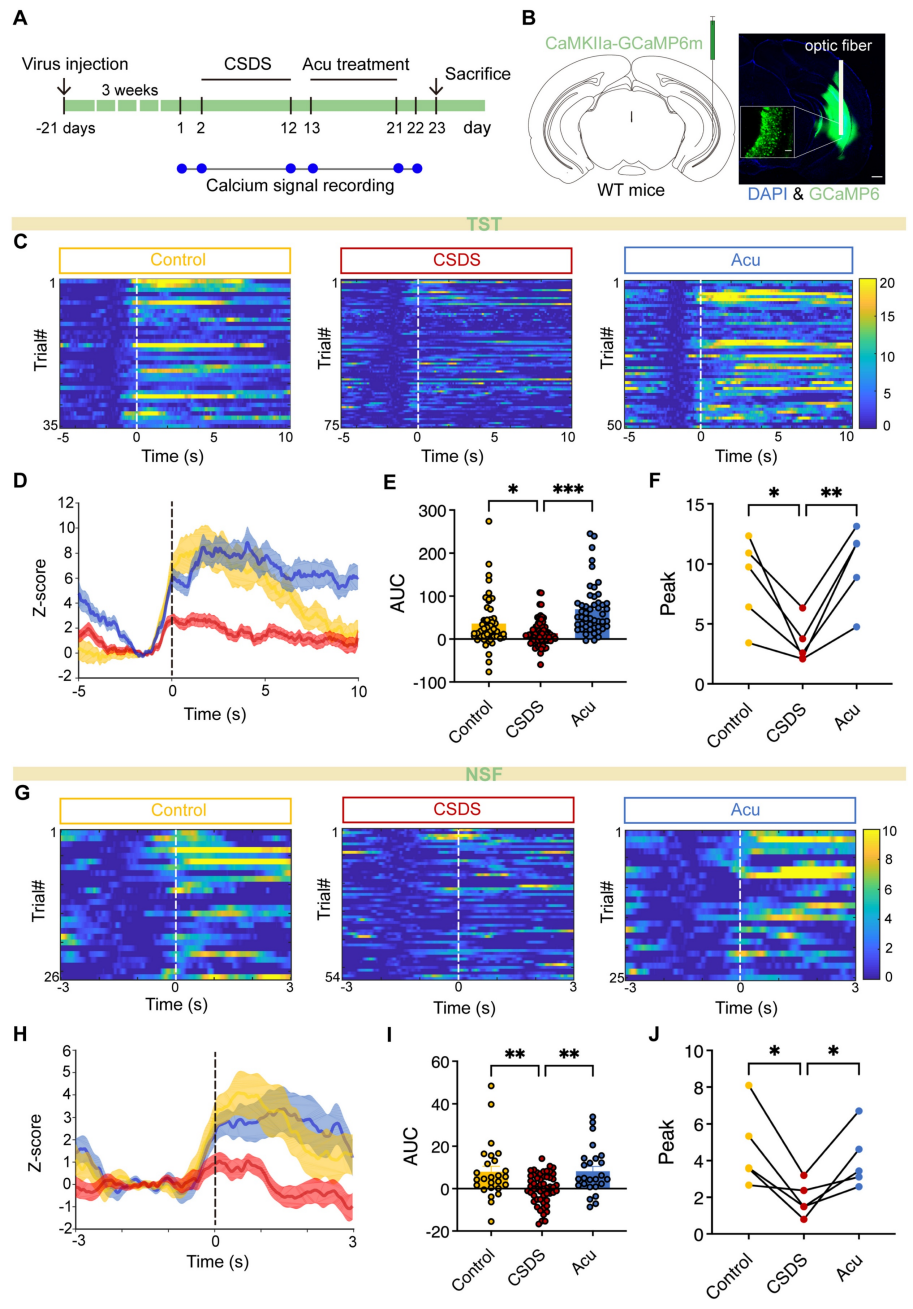
** Acu treatment enhances vCA1 excitatory neuron activity during TST and NSFT. (A)** Timeline of vCA1 calcium signal detection. **(B)** Representative image showing AAV delivered GCaMP6m expression and optical fiber placement in the vCA1; scale bar: 500 μm (main), 50 μm (inset, 20x). **(C)** Heatmap of Ca^2+^ signals from CaMKIIα-positive neurons aligned to the onset of struggling. (n = 35, 75, 50 trials) **(D)** Peri-event plots of Ca^2+^ signals changes from CaMKIIα-positive neurons during Control (yellow), CSCS (red) and Acu (blue) epochs. **(E)** The area under the curve of calcium activity of vCA1 CaMKIIα-positive neurons (Ordinary one-way ANOVA with Dunnett's multiple comparisons test). **(F)** Quantification of TST-induced peak for Control, CSDS and Acu mice (RM one-way ANOVA with Dunnett's multiple comparisons test). **(G)** Heatmap of Ca^2+^ signals from CaMKIIa-positive neurons aligned to the onset of Sniffing food. (n = 26, 54, 25 trials) **(H)** Peri-event plots of Ca^2+^ signals changes from CaMKIIα-positive neurons during Control (yellow), CSCS (red) and Acu (blue) epochs. **(I)** The area under the curve of calcium activity of vCA1 CaMKIIα-positive neurons (Ordinary one-way ANOVA with Dunnett's multiple comparisons test). **(J)** Quantification of NSFT-induced peak for Control, CSDS and Acu mice (RM one-way ANOVA with Dunnett's multiple comparisons test). n = 5 mice/group. Solid lines indicate mean and shaded areas indicate SEM. *P < 0.05, **P < 0.01, ***P < 0.001.

Previous research has mainly focused on the mechanisms underlying Acu's antidepressant effects, with limited attention given to its impact at the brain and circuit levels. To investigate whether Acu treatment modulates the real-time activity of pyramidal neurons during depression-related behaviors and social interaction, we infused an AAV-CaMKIIα-GCaMP6m into the vCA1. This enabled us to analyze neuronal activity changes in freely moving mice using fiber photometry (Figure 3A). GCaMP6m was precisely expressed in CaMKIIα-positive neurons, and the placement of the optical fiber was verified postmortem (Figure 3B). We discovered that pyramidal neuron activity significantly increased during periods of struggling behavior in the TST (Figure 3C). However, in CSDS mice, this activity was significantly reduced during the TST (Figure 3C-F). Remarkably, Acu treatment reversed these deficits (Figure 3C-F), showing a negative correlation between calcium signals and the immobility time (Figure S5). Furthermore, during the NSFT exploration, Ca^2+^ transients were precisely time-locked to the initiation of food-sniffing behavior following the feeding latency period. Excitatory neuron activity was significantly reduced in CSDS mice but substantially increased following Acu treatment (Figure 3G-J). These results suggest that Acu treatment may be associated with enhanced activity of excitatory neurons in the vCA1, which could potentially contribute to its mood-elevating effects.

### Acu treatment regulates synaptic transmission in vCA1 pyramidal neurons

**Figure 4:**
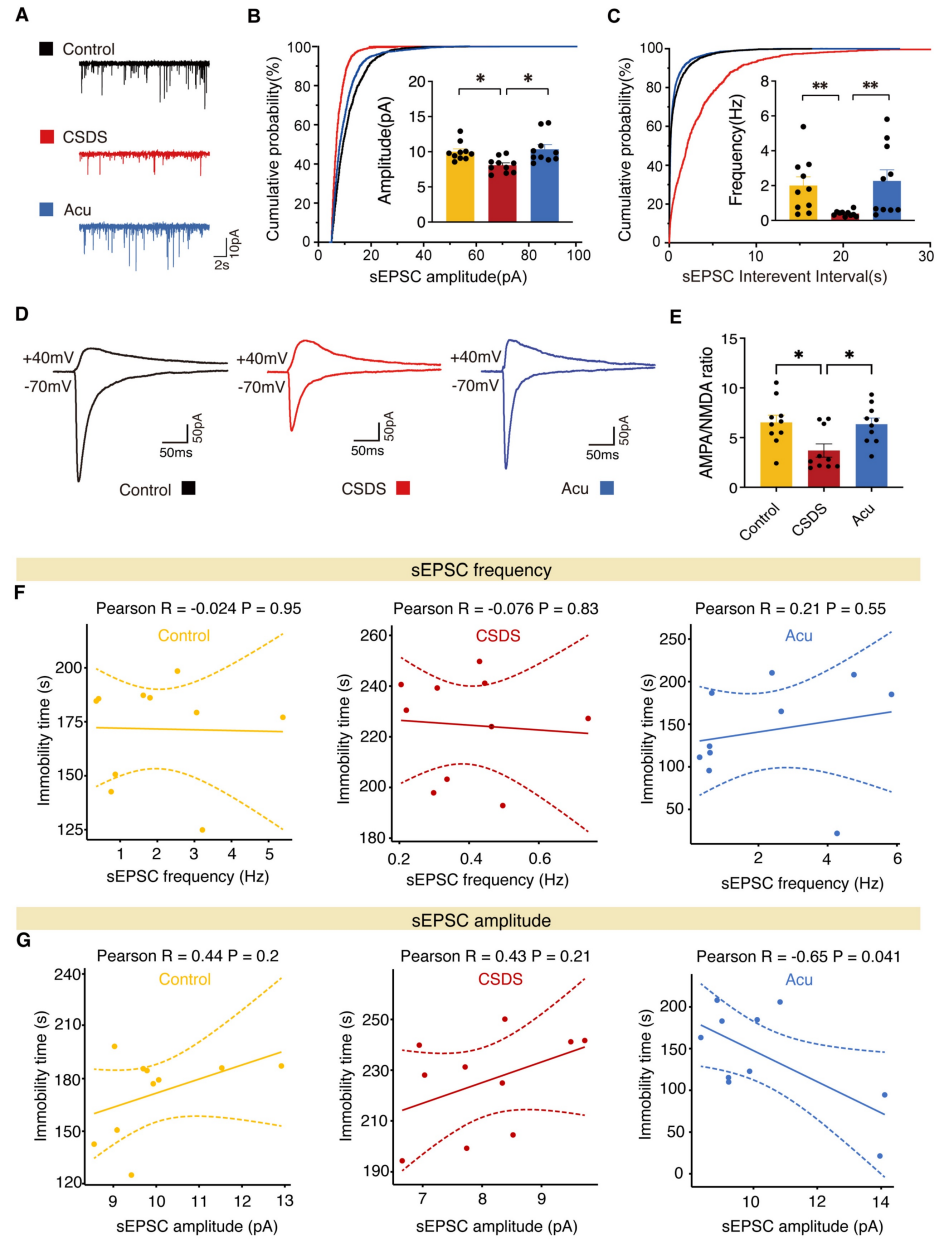
** Acu treatment increases excitatory transmission in vCA1 pyramidal neurons. (A)** Representative traces of sEPSCs recorded from vCA1 pyramidal neurons. **(B, C)** Average sEPSCs amplitude (B) and frequency (C) in Control (yellow), CSDS (red) and Acu (blue) groups, n = 10 cells from 3 mice/group (For all figures: Kolmogorov-Smirnov test). **(D)** Representative traces of AMPAR EPSCs recorded at -70 mV and dual component EPSC at +40 mV.** (E)** AMPA/NMDA ratio is increased by Acu treatment. n = 10 cells from 3 mice/group (For all figures: one-way ANOVA with Tukey's multiple comparisons test).** (F)** Correlations between sEPSCs frequency in vCA1 pyramidal neurons and TST in control, CSDS and Acu mice, respectively. **(G)** Correlations between sEPSCs amplitude in vCA1 pyramidal neurons and TST in control, CSDS and Acu mice, respectively. Data are represented as the mean ± SEM. *P < 0.05, **P < 0.01.

To investigate the role of Acu treatment in synaptic transmission, we performed whole-cell patch-clamp on pyramidal neurons in vCA1 hippocampal coronal slices. Mice exposed to CSDS exhibited a significant decrease in both the amplitude and frequency of spontaneous excitatory postsynaptic currents (sEPSCs), which were reversed by Acu treatment (Figure 4A-C). We next assayed synaptic transmission using AMPAR EPSC to NMDAR EPSC ratios. Following exposure to CSDS, Acu treatment blocked the reduction of the AMPA/NMDA ratio compared with controls (Figure 4D, E). We also examined whether the spontaneous inhibitory postsynaptic current (sIPSCs) were altered (Figure S6A-C). The amplitude of sIPSCs was significantly increased in the CSDS mice group compared with the control group, and there was a trend toward decrease following Acu treatment (Figure S6A, B), although no significant differences were observed in the frequency of sIPSCs among the groups (Figure S6A, C).

Given that Acu treatment enhances glutamatergic transmission, we next examined whether these effects were associated with changes in depression-like behavior. The results indicated that the sEPSCs frequency in the three groups did not correlate with the immobilization time during TST (Figure 4F). Similarly, in both control and CSDS mice, sEPSCs amplitude was not correlated with immobilization time in the TST. Moreover, sIPSCs amplitude did not correlate with the immobilization time during TST in the three groups (Figure S6D). Interestingly, in the Acu mice, sEPSCs amplitude was significantly negatively correlated with immobilization time in the TST (Figure 4G). Collectively, these data suggest that the antidepressant effects of Acu treatment are strongly associated with an increase in postsynaptic AMPAR-driven excitability in vCA1 pyramidal neurons.

### Acu treatment restores synaptic plasticity and BDNF expression impaired by CSDS in the hippocampus

**Figure 5:**
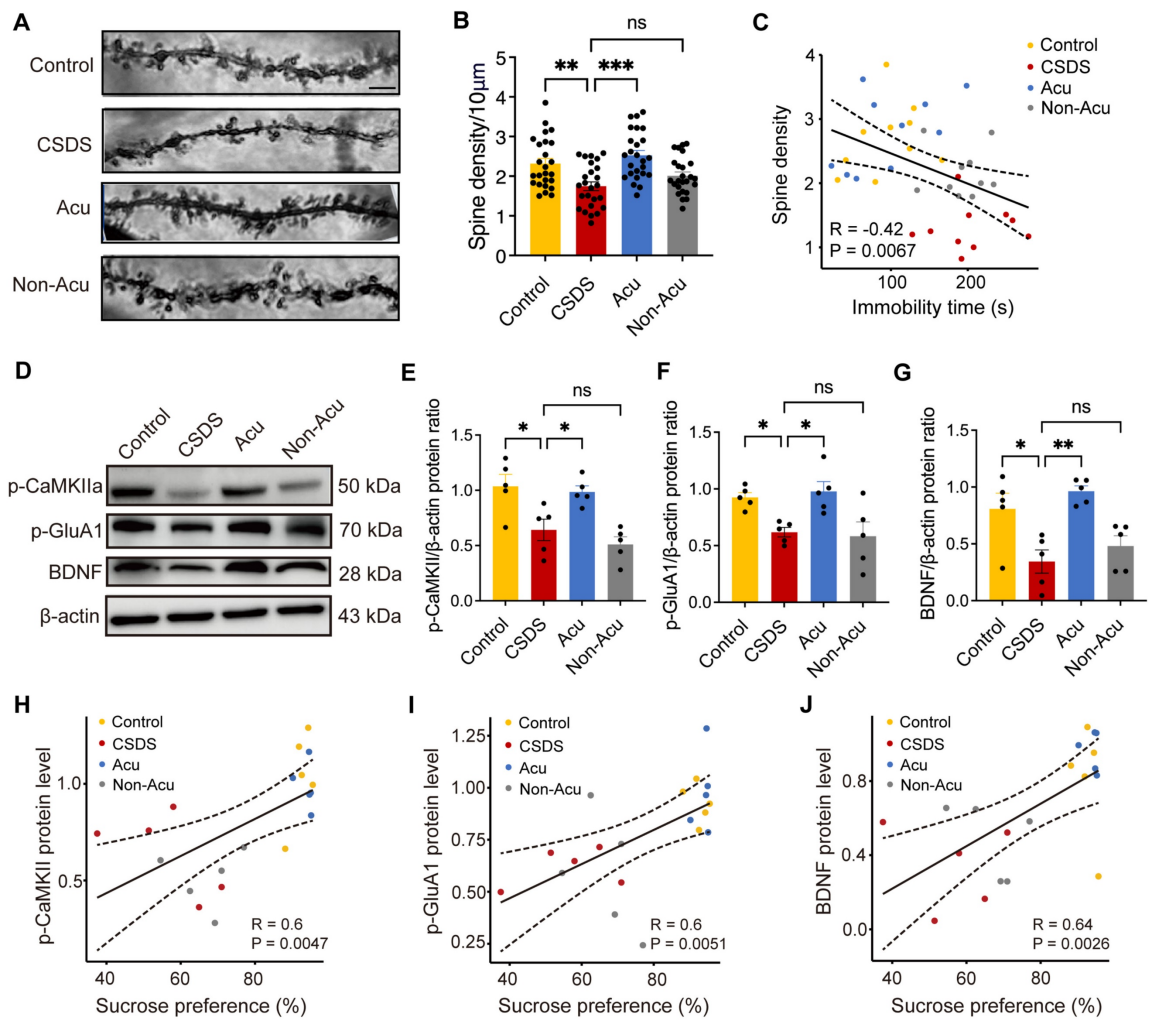
** Acu treatment restores synaptic plasticity and BDNF expression impaired by CSDS in the hippocampus. (A)** The images of apical dendrites in the vCA1 by Golgi stain. Scale bar, 2 μm. **(B)** Quantification of spine numbers, n = 25 dendrites from 5 mice/group (Ordinary one-way ANOVA with Dunnett's multiple comparisons test). **(C)** Correlations between spine density in vCA1 pyramidal neurons and FST. **(D)** Representative immunoblots of p-CaMKII, p-GluA1 and BDNF in hippocampal extracts. **(E-G)** Quantification of p-CaMKII (E), p-GluA1 (F) and BDNF (G), n = 5 mice/group (Ordinary one-way ANOVA with Dunnett's multiple comparisons test). **(H-J)** Correlations between p-CaMKII (H), p-GluA1 (I) and BDNF (J) protein level in hippocampus and SPT. Data are represented as the mean ± SEM. *P < 0.05, **P < 0.01, ***P < 0.001. n.s., no significant difference.

Long-term chronic stress leads to changes in synaptic plasticity in brain regions related to emotions, including decreased complexity and density of neuronal dendrites and dendritic spines, as well as impaired synaptic plasticity 26-28. To visualize spine remodeling before and after CSDS and Acu treatment, we employed Golgi staining (Figure 5A). Data revealed that CSDS significant decreased spine number by approximately 24%, which were reversed after Acu treatment (Figure 5B). Furthermore, a strong negative correlation was observed between immobility time and dendritic spine density in vCA1 pyramidal neurons across the four groups in the FST (Figure 5C). These findings suggest that CSDS reduces unitary excitatory synaptic events by decreasing the number of excitatory synapses, an effect that can be reversed by Acu treatment.

To validate the potential molecular mechanisms underlying the effects of Acu treatment, as predicted by electrophysiology data and Golgi staining, we performed WB assays to detect the expression of phosphorylated CaMKIIα (p-CaMKII), phosphorylated GluA1 (p-GluA1) and phosphorylated GluA2 (p-GluA2). The results indicated that CSDS significantly reduced the expression levels of p-CaMKII and p-Glu A1, while increasing p-GluA2 levels. Acu treatment restored p-CaMKII (Figure 5E), p-GluA1 (Figure 5F) and p-GluA2 (Figure S7B) expression to control levels. p-GluA2 exhibited a negative correlation with the ratio of sucrose preference (Figure S7C), although no significant correlation was observed with immobility time in the TST (Figure S7D). Additionally, the expression of p-CaMKII and p-GluA1 was strongly and positively correlated with the ratio of sucrose preference in the SPT (Figure 5H-J) and negatively correlated with immobility time in the TST (Figure S8A, B). Importantly, Acu treatment restored their expression levels, suggesting that Acu treatment enhances AMPAR trafficking to the cell membrane, potentially via the CaMKII signaling pathway.

Moreover, stress significantly reduced BDNF expression in the hippocampus, leading to impaired synaptic plasticity. We further examined the expression levels of BDNF in hippocampus and observed a restorative effect of Acu treatment and found the proportional reduction of BDNF induced by CSDS was significantly reversed by Acu treatment (Figure 5G). The expression levels of BDNF showed positively correlated with the ratio of sucrose preference in the SPT (Figure 5J) and negatively correlated with immobility time in the TST (Figure S8C). We speculate that the mechanism by which Acu treatment enhances the plasticity of hippocampal pyramidal neurons may involve increased AMPAR trafficking to the cell membrane and enhanced BDNF expression.

## Discussion

In this study, we demonstrated that acupuncture at LR3 effectively alleviates depression-like behaviors induced by CSDS in mice. By utilizing the CSDS model, we closely mimic the symptoms of depression observed in humans, characterized by anhedonia and social avoidance behaviors 21,29,30. Our findings highlight the potential of acupuncture as a therapeutic strategy for managing depression, particularly in individuals who may not respond well to conventional pharmacological treatments.

Calcium signaling, as a major second messenger in the central nervous system, is critical for regulating neuronal excitability and the efficiency of synaptic transmission 31,32. The accumulation of calcium activates various signal pathways that trigger adaptive neuronal responses 33. In addition, calcium concentration in pyramidal neurons expressing CaMKIIα plays a key role in synaptic plasticity 34. Therefore, investigating the alterations in calcium signaling associated with depression is essential. In our study on the mechanism of action of acupuncture at LR3, we employed GCaMP6m-based fiber-optic photometry for the first time to monitor Ca^2+^ transients in vCA1 during the TST, NSFT behavioral tests. Changes in the amplitude of the Ca^2+^ transients responded to increases or decreases in cellular activity in specific regions 35,36. Our observations indicated that cellular activity in vCA1 was closely linked to depression- associated behaviors, such as struggling and food-sniffing.

One key observation in this study was the significant restoration of synaptic structure and function in the hippocampus following Acu treatment. The hippocampus is known to play a crucial role in mood regulation and cognitive function, and its impairment has been linked to the pathophysiology of depression. Neurons in this region are particularly sensitive to stress and undergo remodeling in response to stressors 37. Stress not only induces dendritic atrophy in the CA3 region but also leads to a loss of neuronal spines in the CA1 region 26,38. Our results showed that Acu treatment effectively reversed the apical dendritic atrophy and the reduction in postsynaptic spines density typically associated with CSDS. This suggests that acupuncture may enhance synaptic plasticity, which is critical for mood regulation.

Depression is a complex disorder involving various brain regions and circuits. Recent studies by Ma Qiufu's team 39 have revealed that acupuncture stimulation at the Zusanli (ST36) point activates PROKR2-expressing neurons in peripheral sensory nerves, transmitting signals through the dorsal horn of the spinal cord to the vagal-adrenal axis, which triggers the release of anti-inflammatory mediators like norepinephrine, epinephrine, and dopamine. Although LR3 stimulation activates different peripheral nerves (e.g., the deep peroneal nerve), it may share a similar "spinal cord-brainstem-neurotransmitter" signaling pathway. We hypothesize that LR3 stimulation might activate the spinal cord-PVN pathway, further modulating emotion-related brain regions (e.g., the hippocampus) through glutamatergic projections from the PVN, thereby mediating its antidepressant effects. Previous studies have demonstrated that electroacupuncture at the LR3 point for seven days can improve CRS-induced depressive-like behavior, potentially though the activation of the basolateral amygdala (BLA) 40. Furthermore, it has been suggested that this treatment may exert antidepressant-like effects by activating the nucleus accumbens (NAc) 7. In the present study, we focused on the activation of distinct subregions of the hippocampus, an emotion-related brain region, after CSDS and acupuncture treatment using c-Fos immunofluorescence staining (Figure 2). Our data reveal a significant correlation between the restoration of vCA1 neuronal excitability and behavioral improvement, such as the calcium signal-immobility time correlation in Figures 3. Although our experimental design does not provide direct evidence of causality, the spatiotemporal specificity of vCA1 activation (relative to dCA1 and DG) and its association with the recovery AMPAR and BDNF expression strongly suggest a mechanistic role of vCA1 in mediating antidepressant effects. Future studies incorporate nerve tracing technology could more directly investigate whether hippocampal activation is causally related to the antidepressant effects of acupuncture, providing further clarification on whether hippocampal activation is a key mediator of therapeutic effects or merely an associated response, thus offering a more comprehensive understanding of the underlying mechanisms.

**Figure 6:**
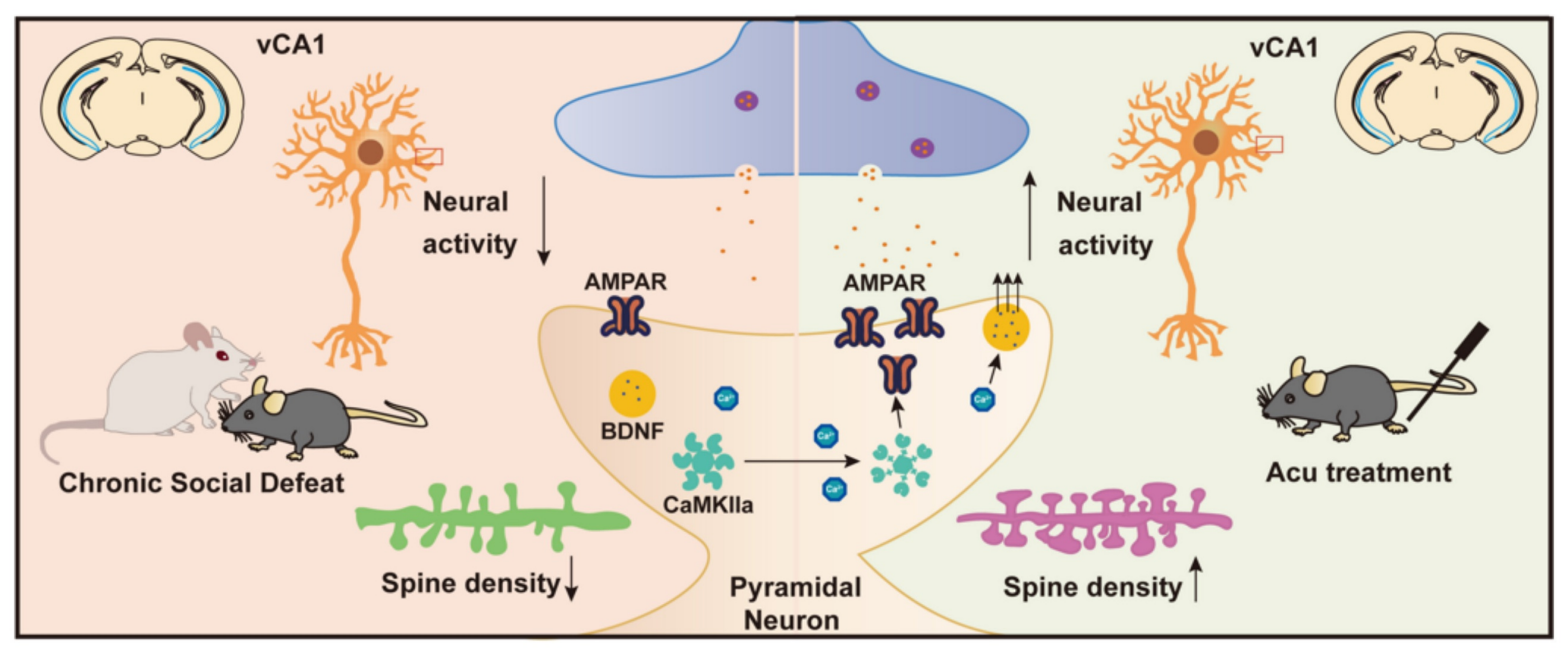
** Schematic summary of present study.** CSDS induces depression-like behaviors in adult mice, accompanied by reduced intrinsic excitability of vCA1 pyramidal neurons and downregulation of synaptic plasticity. Acupuncture (Acu) treatment ameliorates these deficits, and its mechanism may be related to enhancing the membrane trafficking of AMPAR and upregulating the expression of BDNF.

Complex molecular protein interactions play a vital role in regulating synapse formation and neuroplasticity 41. Abnormal expression levels of functional proteins such as AMPAR-associated proteins (e.g., GluA1), postsynaptic dense protein 95 (PSD-95), CaMKII and BDNF, are strongly associated with susceptibility to depression. Our results indicated that acupuncture at LR3 increased GluA1 trafficking to the cell membrane and enhanced BDNF expression, subsequently improving synaptic plasticity (Figure 6). GluA1 is an essential subunit of AMPA receptors, which mediate excitatory neurotransmission and play a vital role in synaptic plasticity. Researchers have found that GluA1 expression is reduced and dephosphorylated, and this dephosphorylation of AMPARs is associated with long-term depression 42. Notably, CSDS significantly reduced the level of GluA1 Ser845 phosphorylation (pSer845) in the hippocampus 43. Acu's ability to downregulate p-GluA2 likely facilitates GluA1/GluA2 heteromer stabilization at the synapse, enabling CaMKII-mediated phosphorylation of GluA1 to insertion into the postsynaptic membrane, enhancing synaptic strength. This mechanism aligns with our observations of restored AMPAR-mediated currents and spine density after Acu treatment. In neurotransmission, CaMKII is phosphorylated at the phosphorylation site Ser831 on GluA1, enhancing electrical conductance through the GluA1 receptor 44. Thus, effective synaptic transmission of AMPARs requires phosphorylation of CaMKII. Furthermore, the elevation of BDNF levels post-treatment underscores the neurotrophic factor's role in promoting neuronal growth and synaptic stabilization. BDNF is well-established as a key player in neuroplasticity 45, and its reduction has been linked to depression 18. It regulates neuroplasticity and synaptic transmission by increasing intercellular Ca²⁺ levels and enhancing mitochondrial motility in neurons. The total BDNF gene expression is significantly downregulated in the hippocampus of socially stressed mice 46-50. The restoration of CaMKII, GluA1, GluA2 and BDNF expression in the hippocampus following Acu treatment suggests an enhancement of AMPAR-mediated synaptic excitation, which may contribute to the observed improvements in depressive behaviors. Although many previous studies have found acupuncture LR3 acupoints to be clinically effective in the treatment of depression 51, further research is necessary to explore other possible mechanisms and assess the effects of other acupuncture points on depression in humans and their potential mechanisms.

In conclusion, our study provides compelling evidence that acupuncture can reverse CSDS-induced depression-like behaviors by enhancing synaptic plasticity and promoting the expression of key neurotrophic factors in the hippocampus. These findings not only support the use of acupuncture as a viable treatment for depression but also pave the way for further research into the underlying mechanisms that govern its therapeutic effects. As the prevalence of depression continues to rise, exploring alternative and complementary treatments like acupuncture may offer valuable options for individuals seeking relief from this debilitating condition.

## Supplementary Material

Supplementary figures and tables.

## Abbreviations

Acu: Acupuncture
vCA1: ventral CA1
CSDS: chronic social defeat stress
LR3: Taichong point
fMRI: functional magnetic resonance imaging
AMPARs: AMPA receptors
LTP: long-term potentiation
BDNF: brain-derived neurotrophic factor
SPT: sucrose preference test
TST: tail suspension test
FST: forced swim test
OFT: open field test
SIT: social interaction test
NSFT: novelty-suppressed feeding test
Acu treatment: Acupuncture treatment
PBS: phosphate-buffered saline
PFA: paraformaldehyde
ACSF: artificial cerebrospinal fluid
PTX: picrotoxin
KYN: kynurenic acid
AUC: area under the curve
PVDF: polyvinylidene fluoride
ANOVA: analysis of variance
PC: passive coping
AP: action potential
sEPSCs: spontaneous excitatory postsynaptic currents
sIPSCs: spontaneous inhibitory postsynaptic current
p-CaMKII: phosphorylated CaMKIIα
p-Glu A1: phosphorylated GluA1
p-Glu A2: phosphorylated GluA2
NAc: nucleus accumbens
LHb: lateral habenula
n.s.: no significance
