# Supplementary material for: Acupuncture alleviates CSDS-induced depressive-like behaviors by modulating synaptic plasticity in vCA1
Source: Theranostics. 2025 Mar 31;15(10):4808–22. doi: 10.7150/thno.106751 (PMC11984413; doi:10.7150/thno.106751)
Supplement: Supplementary file 1 — Supplementary figures and tables. [file thnov15p4808s1.pdf]

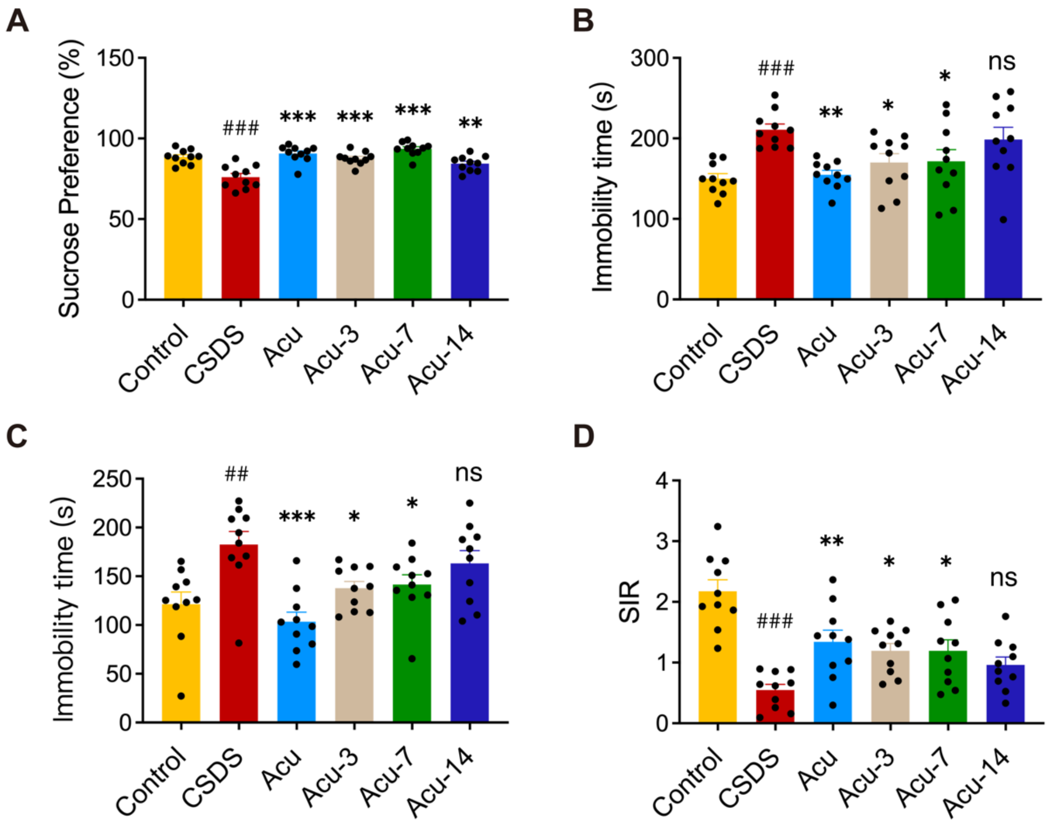

Figure S1. The durability of Acu's therapeutic effects.

(A) Coefficient of sucrose preference in the SPT. **(B)** The duration of immobility in the TST for 5 min. **(C)** The duration of immobility in the FST for 5 min. **(D)** Social Interaction Ratio for the SIT. n = 10 mice/group. One-way ANOVA with Dunnett's multiple comparisons test. Data are represented as the mean ± SEM. Compared with Control group $^{\#\#}P < 0.01$, $^{\#\#\#}P < 0.001$; Compared with CSDS group $^{*}P < 0.05$, $^{**}P < 0.01$, $^{***}P < 0.001$. n.s., no significant difference.

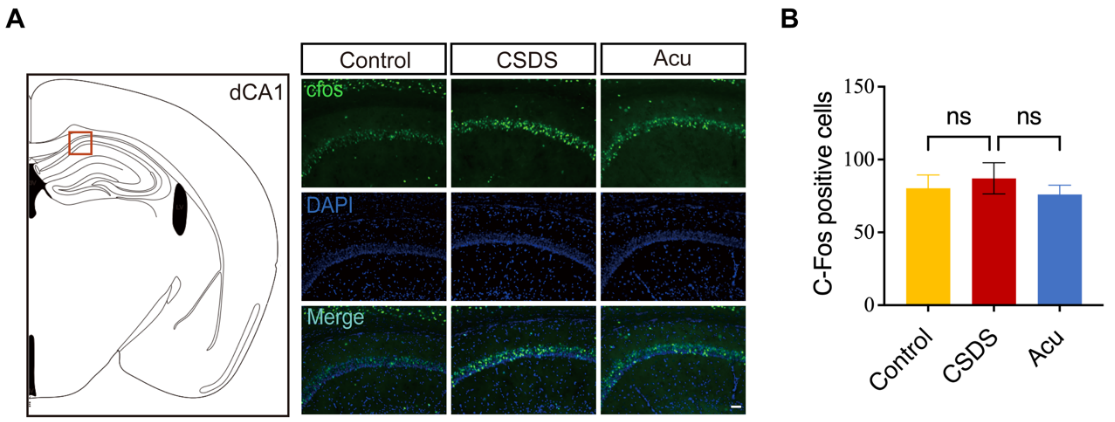

Figure S2. c-Fos expression in dCA1.

(A) The c-Fos expression in dCA1 area. Scale bar, 100 μm. **(B)** Statistical results of c-Fos positive cells, n = 6 mice/group. Ordinary one-way ANOVA with Dunnett's multiple comparisons test. Data are represented as the mean ± SEM. n.s., no significant difference.

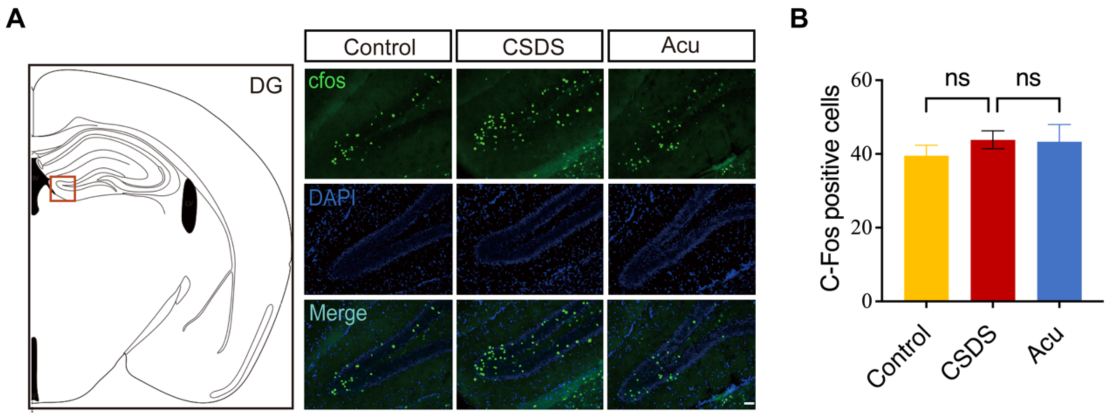

Figure S3. c-Fos expression in DG.

(A) The c-Fos expression in DG area. Scale bar, 100 μm. **(B)** Statistical results of c-Fos positive cells, n = 3 mice/group. Ordinary one-way ANOVA with Dunnett's multiple comparisons test. Data are represented as the mean ± SEM. n.s., no significant difference.

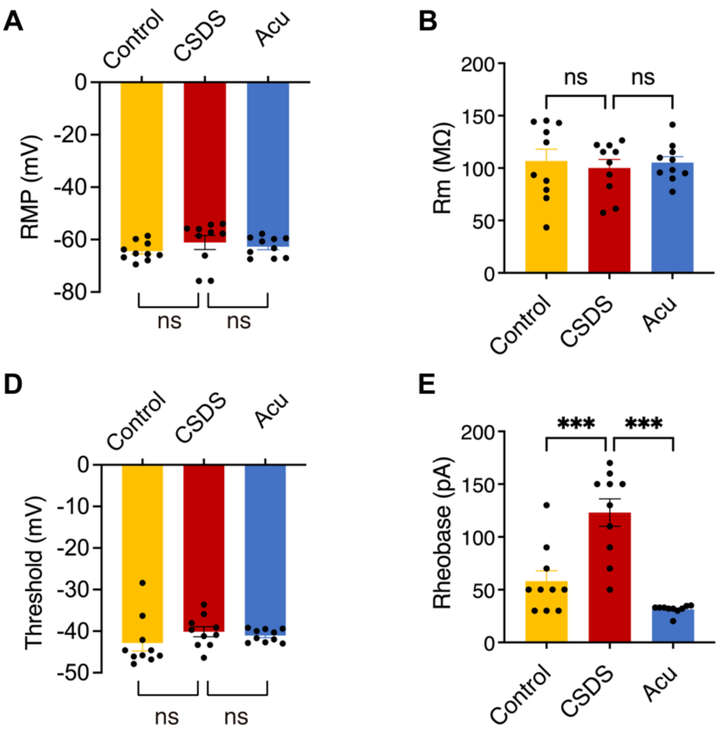

Figure S4. Electrophysiological properties of vCA1 pyramidal neurons under CSDS and Acu treatment

(A) Resting membrane potential (RMP) of pyramidal neurons in vCA1. **(B)** Membrane resistance (Rm) of pyramidal neurons in vCA1. **(C)** Membrane capacitance (Cm) of pyramidal neurons in vCA1. **(D)** Threshold of pyramidal neurons in vCA1. **(E)** Rheobase of pyramidal neurons in vCA1. n = 10 neurons from 3 mice/group, respectively. One-way ANOVA with Tukey's multiple comparisons test. Data are represented as the mean ± SEM. ***P<0.001. n.s., no significant difference.

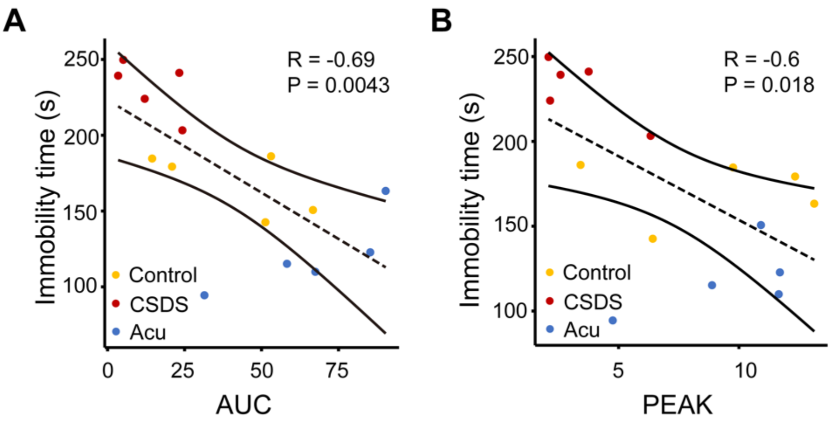

Figure S5. Correlations between activity of vCA1 pyramidal neurons and TST

(A) Correlations between the activity of vCA1 pyramidal neurons and the immobility time in the TST (Pearson correlation coefficient, R = -0.69, P = 0.0043). **(B)**

Correlations between PEAK in vCA1 pyramidal neurons and the immobility time in the TST (Pearson correlation coefficient, R = -0.6, P = 0.018).

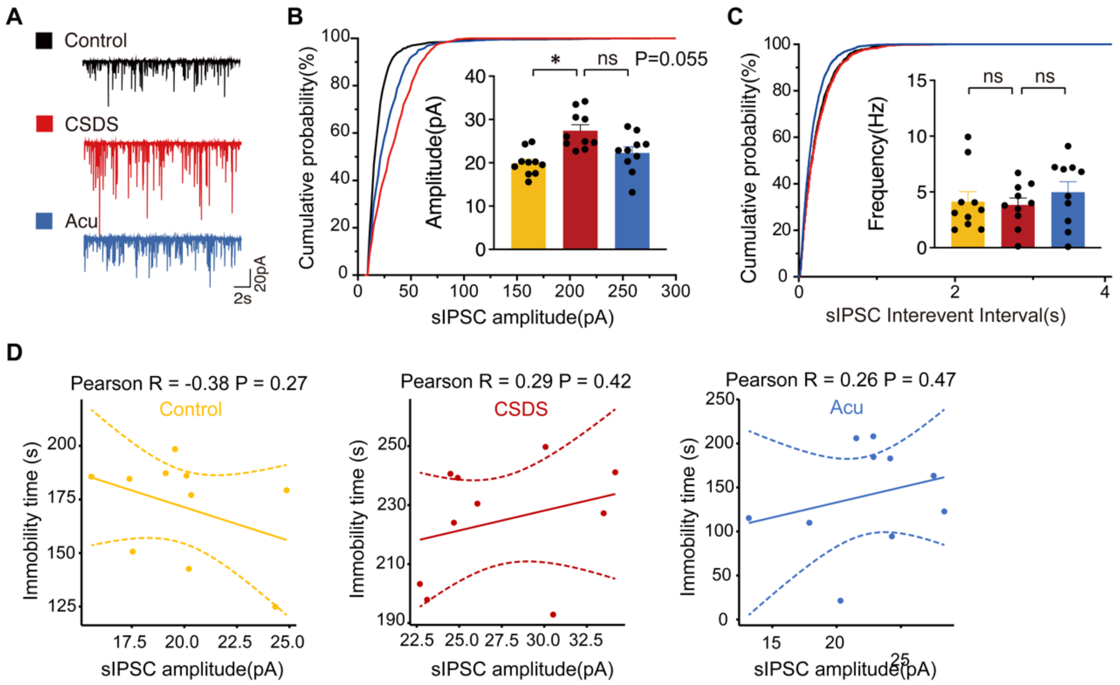

Figure S6. Inhibitory transmission in vCA1 pyramidal neurons.

(A) Representative traces of sIPSCs recorded from vCA1 pyramidal neurons. **(B, C)** Average sIPSCs amplitude (B) and frequency (C) in Control (yellow), CSDS (red) and Acu (blue) groups. n = 10 cells from 3 mice/group (For all figures: Kolmogorov-Smirnov test). **(D)** Correlations between sIPSC amplitude in vCA1 pyramidal neurons and TST in control, CSDS and Acu mice, respectively. Data are represented as the mean ± SEM. *P < 0.05, n.s., no significant difference.

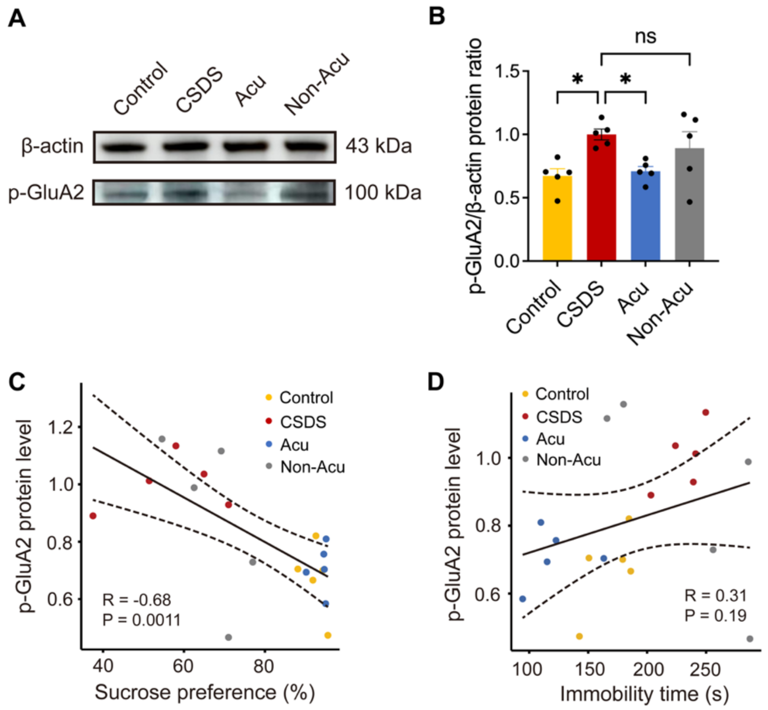

Figure S7. p-GluA2 expression induced by Acu treatment in the hippocampus

(A) Representative immunoblots of p-GluA2 in hippocampal extracts. **(B)** Quantification of p-GluA2, n = 5 mice/group (Ordinary one-way ANOVA with Dunnett's multiple comparisons test). **(C)** Correlations between p-GluA2 protein level in hippocampus and SPT (Pearson correlation coefficient, R = -0.68, P = 0.0011). **(D)** Correlations between p-GluA2 protein level in hippocampus and TST (Pearson correlation coefficient, R = 0.31, P = 0.19). Data are represented as the mean ± SEM. *P < 0.05, n.s., no significant difference.

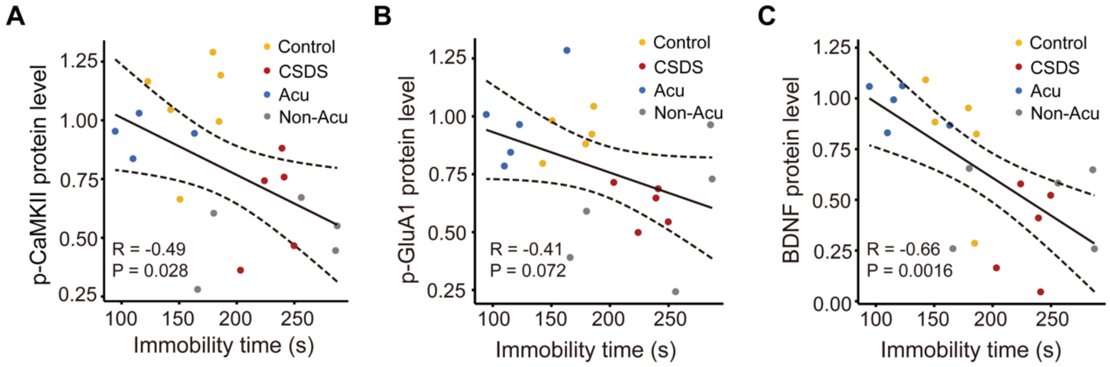

Figure S8. Correlations between protein levels in hippocampus and TST

(A) Correlations between pCaMKII protein level in hippocampus and TST (Pearson correlation coefficient, R = -0.49, P = 0.028). **(B)** Correlations between p-GluA1 protein level in hippocampus and TST (Pearson correlation coefficient, R = -0.41, P = 0.072). **(C)** Correlations between BDNF protein level in hippocampus and TST (Pearson correlation coefficient, R = -0.66, P = 0.0016).

Supplementary Table 1

Statistics reporting, by figure

		Sample size (figure order)	Statistical test	Treatment effect		Significance
**Figure 1**						
**B**	SPT of three groups	n = 10 mice/group.	One-way ANOVA (Tukey's multiple comparisons test)	F (3, 36) = 3.160	P = 0.036	P < 0.05
	Control vs. CSDS				P = 0.002	P < 0.01
	CSDS vs. Acu				P < 0.001	P < 0.001
	CSDS vs. Non-Acu				P = 0.593	n.s.
**C**	TST of three groups	n = 10 mice/group.	One-way ANOVA (Tukey's multiple comparisons test)	F (3, 36) = 4.860	P = 0.006	P < 0.01
	Control vs. CSDS				P = 0.022	P < 0.05
	CSDS vs. Acu				P < 0.001	P < 0.001
	CSDS vs. Non-Acu				P = 0.963	n.s.
**D**	FST of three groups	n = 10 mice/group.	One-way ANOVA (Tukey's multiple comparisons test)	F (3, 36) = 0.9457	P = 0.429	n.s.
	Control vs. CSDS				P < 0.001	P < 0.001
	CSDS vs. Acu				P < 0.001	P < 0.001
	CSDS vs. Non-Acu				P = 0.938	n.s.
**F**	SIR of three groups	n = 10 mice/group.	One-way ANOVA (Tukey's multiple comparisons test)	F (3, 36) = 2.857	P = 0.050	n.s.
	Control vs. CSDS				P < 0.001	P < 0.001
	CSDS vs. Acu				P = 0.030	P < 0.05
	CSDS vs. Non-Acu				P = 0.562	n.s.
**G**	Interaction time of three groups	n = 10 mice/group.	Two-way ANOVA (Tukey's multiple comparisons test)	F (3, 72) = 5.647	P = 0.002	P < 0.01
	No target: Control vs. No target: CSDS				P > 0.999	n.s.
	No target: CSDS vs. No target: Acu				P = 0.999	n.s.
	No target: CSDS vs. No target: Non-Acu				P > 0.999	n.s.
	No target: Control vs. Target: Control				P = 0.027	P < 0.05
	Target: Control vs. Target: CSDS				P < 0.001	P < 0.001
	Target: CSDS vs. Target: Acu				P = 0.965	n.s.
	Target: CSDS vs. Target: Non-Acu				P = 0.990	n.s.

**I**	OFT of three groups	n = 10 mice/group.	One-way ANOVA (Tukey's multiple comparisons test)	F (3, 36) = 0.4390	P = 0.726	n.s.
	Control vs. CSDS				P = 0.973	n.s.
	CSDS vs. Acu				P = 0.908	n.s.
	CSDS vs. Non-Acu				P = 0.861	n.s.
**J**	OFT of three groups	n = 10 mice/group.	One-way ANOVA (Tukey's multiple comparisons test)	F (3, 36) = 1.284	P = 0.294	n.s.
	Control vs. CSDS				P = 0.800	n.s.
	CSDS vs. Acu				P = 0.965	n.s.
	CSDS vs. Non-Acu				P = 0.990	n.s.
**Figure 2**						
**D**	c-Fos of three groups	n = 3 mice/group.	One-way ANOVA (Tukey's multiple comparisons test)	F (2, 9) = 15.22	P = 0.001	P = 0.01
	Control vs. CSDS				P = 0.002	P < 0.01
	CSDS vs. Acu				P = 0.004	P < 0.01
**H**	Fring frequency of three groups	n = 10 neurons from 3 mice/group.	One-way ANOVA (Tukey's multiple comparisons test)	F (2, 27) = 21.87	P < 0.001	P < 0.001
	Control vs. CSDS				P < 0.001	P < 0.001
	CSDS vs. Acu				P < 0.001	P < 0.001
**Figure 3**						
**E**	TST AUC of three groups	n = 5 mice/group.	Ordinary one-way ANOVA (Dunnett's multiple comparisons test)	F (2, 171) = 5.994	P = 0.003	P < 0.01
	CSDS vs. Control				P = 0.020	P < 0.05
	CSDS vs. Acu				P < 0.001	P < 0.001
**F**	TST PEAK of three groups	n = 5 mice/group.	Ordinary one-way ANOVA (Dunnett's multiple comparisons test)	F (1.165, 4.661) = 19.57	P = 0.007	P < 0.01
	CSDS vs. Control				P = 0.040	P < 0.05
	CSDS vs. Acu				P < 0.001	P < 0.001
**I**	NSF AUC of three groups	n = 5 mice/group.	Ordinary one-way ANOVA (Dunnett's multiple comparisons test)	F (2, 102) = 1.975	P = 0.144	n.s.
	CSDS vs. Control				P = 0.003	P < 0.01
	CSDS vs. Acu				P = 0.002	P < 0.01

**J**	NSF PEAK of three groups	n = 5 mice/group.	Ordinary one-way ANOVA (Dunnett's multiple comparisons test)	F (1.714, 6.856) = 9.869	P = 0.11	n.s.
	CSDS vs. Control				P = 0.040	P < 0.05
	CSDS vs. Acu				P = 0.039	P < 0.05
**Figure 4**						
**B**	sEPSC Amplitude of three groups	n = 10 neurons from 3 mice/group.	Kolmogorov-Smirnov test			
	Control vs. CSDS				P = 0.0149	P < 0.05
	CSDS vs. Acu				P = 0.0149	P < 0.05
**C**	sEPSC Frequency of three groups	n = 10 neurons from 3 mice/group.	Kolmogorov-Smirnov test			
	Control vs. CSDS				P = 0.003	P < 0.01
	CSDS vs. Acu				P = 0.003	P < 0.01
**E**	AMPA/NMDA ratio of three groups	n = 10 neurons from 3 mice/group.	One-way ANOVA (Tukey's multiple comparisons test)	F (2, 27) = 5.605	P = 0.009	P < 0.01
	Control vs. CSDS				P = 0.016	P < 0.05
	CSDS vs. Acu				P = 0.024	P < 0.05
**Figure 5**						
**B**	golgi of three groups	n = 5 mice/group.	Ordinary one-way ANOVA (Dunnett's multiple comparisons test)	F (3, 96) = 0.9563	P = 0.417	n.s.
	CSDS vs. Control				P = 0.002	P < 0.01
	CSDS vs. Acu				P < 0.001	P < 0.001
	CSDS vs. Non-Acu				P = 0.242	n.s.
**E**	WB of three groups	n = 5 mice/group.	Ordinary one-way ANOVA (Dunnett's multiple comparisons test)	F (3, 16) = 0.4722	P = 0.706	n.s.
	CSDS vs. Control				P = 0.012	P < 0.05
	CSDS vs. Acu				P = 0.029	P < 0.05
	CSDS vs. Non-Acu				P = 0.568	n.s.
**F**	WB of three groups	n = 5 mice/group.	Ordinary one-way ANOVA (Dunnett's multiple comparisons test)	F (3, 16) = 1.970	P = 0.159	n.s.

	CSDS vs. Control				P = 0.046	P < 0.05
	CSDS vs. Acu				P = 0.018	P < 0.05
	CSDS vs. Non-Acu				P = 0.981	n.s.
**G**	WB of three groups	n = 5 mice/group.	Ordinary one-way ANOVA (Dunnett's multiple comparisons test)	F (3, 16) = 0.4087	P = 0.749	n.s.
	CSDS vs. Control				P = 0.013	P < 0.05
	CSDS vs. Acu				P = 0.001	P < 0.01
	CSDS vs. Non-Acu				P = 0.661	n.s.
**Figure S1.**						
**A**	SPT of five groups	n = 10 mice/group.	Ordinary one-way ANOVA (Dunnett's multiple comparisons test)	F (5, 54) = 14.29	P < 0.001	P < 0.001
	CSDS vs. Control				P < 0.001	P < 0.001
	CSDS vs. Acu				P < 0.001	P < 0.001
	CSDS vs. Acu-3				P < 0.001	P < 0.001
	CSDS vs. Acu-7				P < 0.001	P < 0.001
	CSDS vs. Acu-14				P = 0.002	P < 0.01
**B**	TST of five groups	n = 10 mice/group.	Ordinary one-way ANOVA (Dunnett's multiple comparisons test)	F (5, 54) = 5.072	P < 0.001	P < 0.001
	CSDS vs. Control				P < 0.001	P < 0.001
	CSDS vs. Acu				P = 0.002	P < 0.01
	CSDS vs. Acu-3				P = 0.039	P < 0.05
	CSDS vs. Acu-7				P = 0.048	P < 0.05
	CSDS vs. Acu-14				P = 0.883	n.s.
**C**	FST of five groups	n = 10 mice/group.	Ordinary one-way ANOVA (Dunnett's multiple comparisons test)	F (5, 54) = 6.485	P < 0.001	P < 0.001
	CSDS vs. Control				P = 0.001	P < 0.01
	CSDS vs. Acu				P < 0.001	P < 0.001
	CSDS vs. Acu-3				P = 0.027	P < 0.05
	CSDS vs. Acu-7				P = 0.049	P < 0.05
	CSDS vs. Acu-14				P = 0.620	n.s.
**D**	SIR of five groups	n = 10 mice/group.	Ordinary one-way ANOVA (Dunnett's multiple comparisons test)	F (5, 54) = 11.88	P < 0.001	P < 0.001
	CSDS vs. Control				P < 0.001	P < 0.001

	CSDS vs. Acu				P = 0.003	P < 0.01
	CSDS vs. Acu-3				P = 0.021	P < 0.05
	CSDS vs. Acu-7				P = 0.021	P < 0.05
	CSDS vs. Acu-14				P = 0.231	n.s.
**Figure S2.**	c-Fos of three groups	n = 6 mice/group.	Ordinary one-way ANOVA (Dunnett's multiple comparisons test)	F (2, 33) = 1.141	P = 0.332	n.s.
**C**	CSDS vs. Control				P = 0.814	n.s.
	CSDS vs. Acu				P = 0.590	n.s.
**Figure S3.**	c-Fos of three groups	n = 3 mice/group.	Ordinary one-way ANOVA (Dunnett's multiple comparisons test)	F (2, 15) = 1.617	P = 0.231	n.s.
**B**	CSDS vs. Control				P = 0.591	n.s.
	CSDS vs. Acu				P = 0.992	n.s.
**Figure S4.**						
**A**	RMP of three groups	n = 10 neurons from 3 mice/group.	One-way ANOVA (Tukey's multiple comparisons test)	F (2, 27) = 8.795	P = 0.012	P < 0.05
	Control vs. CSDS				P = 0.404	n.s.
	CSDS vs. Acu				P = 0.816	n.s.
**B**	Rm of three groups	n = 10 neurons from 3 mice/group.	One-way ANOVA (Tukey's multiple comparisons test)	F (2, 27) = 3.757	P = 0.036	P < 0.05
	Control vs. CSDS				P = 0.859	n.s.
	CSDS vs. Acu				P = 0.912	n.s.
**C**	Cm of three groups	n = 10 neurons from 3 mice/group.	One-way ANOVA (Tukey's multiple comparisons test)	F (2, 27) = 0.4162	P = 0.664	n.s.
	Control vs. CSDS				P = 0.878	n.s.
	CSDS vs. Acu				P = 0.986	n.s.
**D**	Threshold of three groups	n = 10 neurons from 3 mice/group.	One-way ANOVA (Tukey's multiple comparisons test)	F (2, 27) = 1.201	P = 0.317	n.s.
	Control vs. CSDS				P = 0.335	n.s.
	CSDS vs. Acu				P = 0.878	n.s.
**E**	Rheobase of three groups	n = 10 neurons from 3 mice/group.	One-way ANOVA (Tukey's multiple comparisons test)	F (2, 27) = 4.988	P = 0.014	P < 0.05
	Control vs. CSDS				P < 0.001	P < 0.001

	CSDS vs. Acu				P < 0.001	P < 0.001
**Figure S6.**						
**B**	sIPSC Amplitude of three groups	n = 10 neurons from 3 mice/group.	Kolmogorov-Smirnov test			
	Control vs. CSDS				P = 0.003	P < 0.01
	CSDS vs. Acu				P = 0.055	n.s.
**C**	sIPSC Frequency of three groups	n = 10 neurons from 3 mice/group.	Kolmogorov-Smirnov test			
	Control vs. CSDS				P = 0.759	n.s.
	CSDS vs. Acu				P = 0.164	n.s.
**Figure S7.**						
	WB of three groups	n = 5 mice/group.	Ordinary one-way ANOVA (Dunnett's multiple comparisons test)	F (3, 16) = 4.086	P = 0.025	P < 0.05
	CSDS vs. Control				P = 0.021	P < 0.05
	CSDS vs. Acu				P = 0.041	P < 0.05
	CSDS vs. Non-Acu				P = 0.630	n.s.
